# In This Issue: Volume 117, Issue 9, September 2026

**DOI:** 10.1111/cas.70501

**Published:** 2026-09-01

## Organ‐Specific Cancer‐Associated Fibroblast Subtypes Across the Digestive System Linked to Cancer Hallmarks

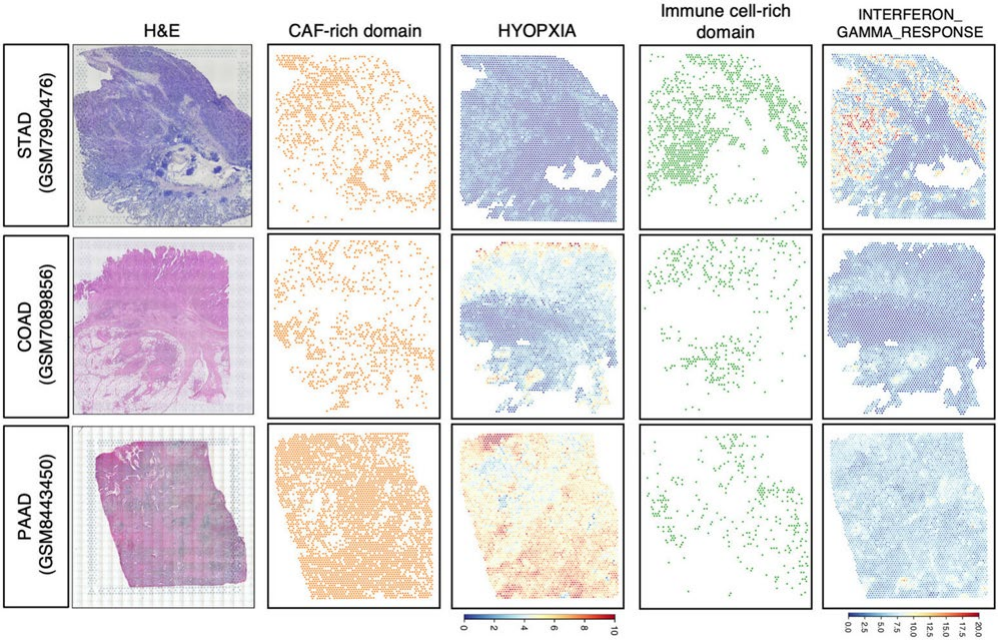

Cancer growth depends not only on cancer cells but also on the surrounding tumor microenvironment, where many cell types interact. Among them are cancer‐associated fibroblasts (CAFs), which can either support or restrain tumors. Since all tumors vary in the abundance and dominance of specific CAF subtypes, overgeneralizing a single CAF classification across different tissue types may be unreliable.

Gastrointestinal (GI) cancers account for almost one‐third of cancer‐related mortality worldwide. Researchers wanted to better understand how different CAF subtypes impact various GI cancers. For this, they analyzed hundreds of tumor samples from stomach, colorectal, pancreatic, and biliary tract cancers. Using single‐cell sequencing, machine learning, and spatial mapping technologies, the team examined where different fibroblast groups were located, how they communicated with neighboring cells, and whether their presence was linked to patient outcomes.

The study identified eight distinct CAF subtypes, each with unique characteristics and patterns across digestive system cancers. Two subtypes, called myofibroblastic CAF (myCAF1 and myCAF2), were consistently linked to poor survival. These cells were associated with tissue remodeling, low‐oxygen adaptation, and close interactions with immune cells that suppress the body's natural defenses against cancer. They also communicated with aggressive cancer cells that are more likely to spread. In contrast, another subtype, known as inflammatory CAF (iCAF1), was associated with better survival. Tumors enriched with iCAF1 contained higher levels of immune cells that can recognize and attack cancer, suggesting a more active anti‐cancer response.

Together, these findings show that CAFs are far more diverse than previously appreciated and that their location and behavior within tumors can strongly influence disease progression. Different GI cancers contained distinct mixtures of fibroblast subtypes, highlighting the importance of tissue‐specific tumor environments. Rather than targeting all fibroblasts indiscriminately, future treatments may benefit from focusing on harmful subtypes while preserving or enhancing those linked to stronger immune responses. This work provides a framework for developing more precise and personalized therapies for digestive system cancers. Such strategies could improve treatment effectiveness while minimizing disruption to beneficial cells within the tumor environment, ultimately supporting better long‐term outcomes for patients.

https://onlinelibrary.wiley.com/doi/full/10.1111/cas.70435

## Oncogenic BRAF and KRAS Promote Global DNA Hypomethylation Through a Directed Pathway That Upregulates TET3

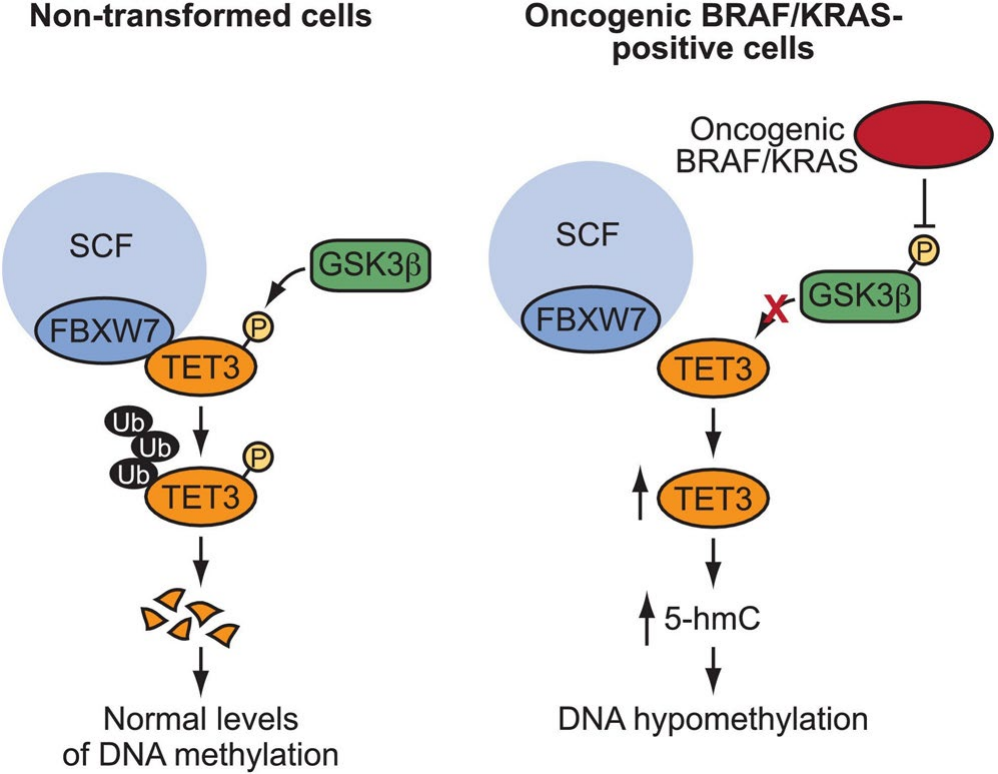

Cancer develops not only because of changes in DNA sequences but also from alterations in the chemical marks that control how genes are switched on and off. One such mark, DNA methylation, usually helps regulate gene expression in healthy cells. While scientists have long understood how excessive DNA methylation can silence protective genes in cancer, far less is known about why cancer cells lose DNA methylation across large regions of their genome. This study explored how two well‐known cancer‐driving genes, BRAF and KRAS, trigger these widespread epigenetic changes during early tumor development.

Using genetically engineered mouse cells and mouse models of lung cancer, the researchers followed the events that occur after cancer‐causing forms of BRAF or KRAS become active. They discovered that these mutations increase the amount of a protein called TET3, which removes DNA methylation marks by converting them into an intermediate form. Normally, another protein, FBXW7, keeps TET3 under control by marking it for destruction after it is modified by GSK3β. However, cancer‐causing BRAF and KRAS block this quality‐control process, allowing TET3 to accumulate and promote widespread DNA hypomethylation. The team also found that reducing TET3 levels lowered tumor formation and increased cancer cell death in mice, suggesting that TET3 helps mutated cells survive during the earliest phases of cancer. Similar molecular patterns were observed in human colorectal precancerous tissues carrying BRAF mutations.

Together, these findings reveal a previously unknown pathway through which cancer‐driving mutations reshape the epigenetic landscape long before tumors become fully established. Rather than being a random event, global DNA hypomethylation appears to result from a coordinated biological process driven by BRAF, KRAS, and TET3. The study suggests that increased TET3 and related DNA changes could serve as early warning signs of cancer development and may provide promising targets for future strategies aimed at preventing or delaying tumor formation.

https://onlinelibrary.wiley.com/doi/full/10.1111/cas.70434

## Targeting Cell Cycle Vulnerabilities in Cancers: Emerging Strategies for Therapeutic Development

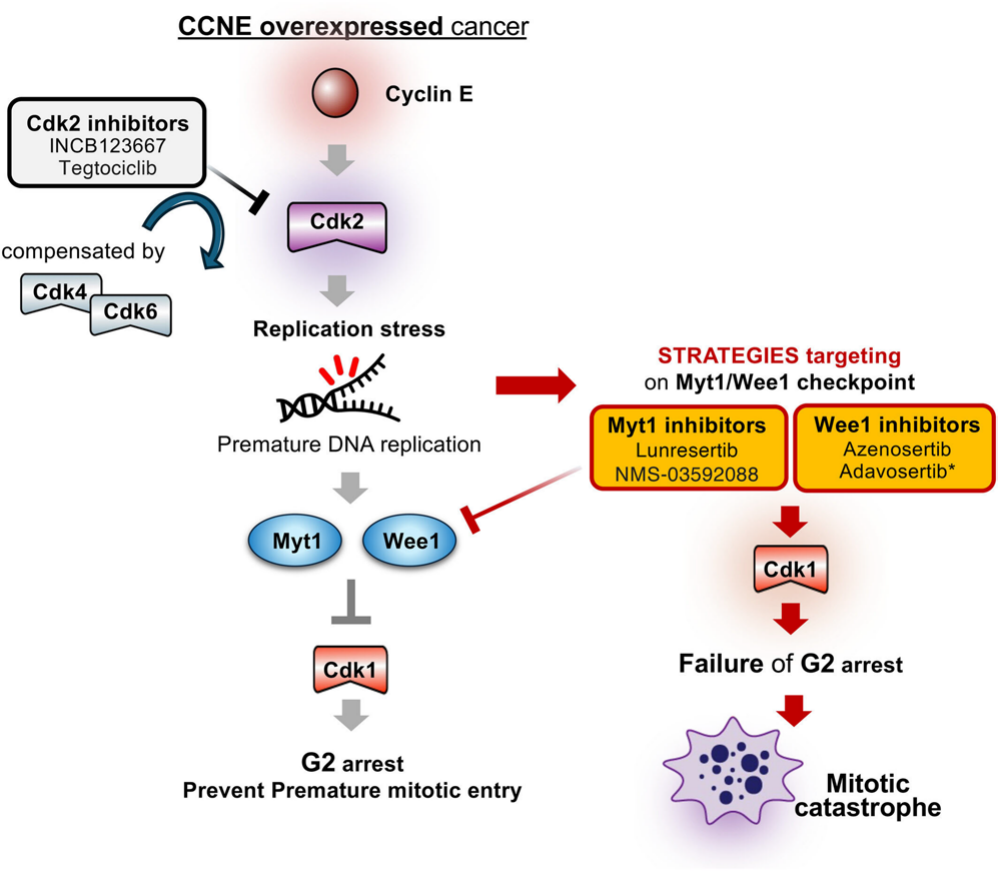

This review summarizes emerging strategies that exploit cancer‐specific vulnerabilities in cell‐cycle regulation and DNA damage responses. Cancer develops when cells lose control over when they should grow and divide. Healthy cells follow a carefully regulated cycle that allows damaged DNA to be repaired before new cells are formed. Cancer cells often bypass these safety checks, which allow them to multiply rapidly and accumulate harmful genetic changes. Although this makes tumors more aggressive, it also forces cancer cells to rely on backup systems that help them survive under constant stress.

The authors highlight several ways cancer cells become vulnerable because they depend on specific proteins that control cell division, repair DNA damage, or pause growth until damage is fixed. Some existing medicines already act on these processes by slowing cell growth or disrupting DNA repair.

Researchers are also exploring approaches that push cancer cells to divide before they are ready, creating fatal errors that healthy cells are better able to avoid. At the same time, gene‐editing technologies are helping scientists find vulnerabilities that exist only in tumor cells. This makes it possible to design targeted therapies that avoid the heavy side effects of standard chemotherapy.

In short, these discoveries prove that the aggressive growth driving cancer also leaves it exposed. Rather than attacking all dividing cells, future therapies could target the survival strategies cancer cells rely on, making treatment more precise and potentially reducing side effects. Mapping these hidden vulnerabilities gives researchers a clear blueprint for targeted treatments, and offers a way to hit tumors where they are most vulnerable without overwhelming the rest of the body.

https://onlinelibrary.wiley.com/doi/full/10.1111/cas.70438

